# Supplementary material for: Modelling Skylarks (Alauda arvensis) to Predict Impacts of Changes in Land Management and Policy: Development and Testing of an Agent-Based Model
Source: PLoS One. 2013 Jun 6;8(6):e65803. doi: 10.1371/journal.pone.0065803 (PMC3675089; doi:10.1371/journal.pone.0065803)
Supplement: Supporting Information S4 — The skylark ODdox as a zipped archive. (ZIP) [file pone.0065803.s004.zip › Skylark_ODdox/struct_farm_event.html]

ALMaSS Skylark ODdox: FarmEvent Struct Reference


ALMaSS Skylark ODdox
 2.0


- Main Page
- Related Pages
- Classes
- Files

- Class List
- Class Index
- Class Hierarchy
- Class Members

Public Member Functions |
Public Attributes

FarmEvent Struct Reference

A struct to hold the information required to trigger a farm event.
More...

`#include <farm.h>`

List of all members.

|  |  |
| --- | --- |
| Public Member Functions | |
|  | FarmEvent (TTypesOfVegetation a\_event, LE \*a\_field, int a\_todo, long a\_run, bool a\_lock, int a\_start, bool a\_first\_year, TTypesOfVegetation a\_nextcrop) |

|  |  |
| --- | --- |
| Public Attributes | |
| TTypesOfVegetation | m\_event |
| LE \* | m\_field |
| bool | m\_first\_year |
| bool | m\_lock |
| TTypesOfVegetation | m\_next\_tov |
| long | m\_run |
| int | m\_startday |
| int | m\_todo |

---

## Detailed Description

A struct to hold the information required to trigger a farm event.

---

## Constructor & Destructor Documentation

|  |  |  |  |
| --- | --- | --- | --- |
| FarmEvent::FarmEvent | ( | TTypesOfVegetation | *a\_event*, |
|  |  | LE \* | *a\_field*, |
|  |  | int | *a\_todo*, |
|  |  | long | *a\_run*, |
|  |  | bool | *a\_lock*, |
|  |  | int | *a\_start*, |
|  |  | bool | *a\_first\_year*, |
|  |  | TTypesOfVegetation | *a\_nextcrop* |
|  | ) |  |  |

inline

References m\_event, m\_field, m\_first\_year, m\_lock, m\_next\_tov, m\_run, m\_startday, and m\_todo.

{

m\_event = a\_event;

m\_field = a\_field;

m\_todo = a\_todo;

m\_run = a\_run;

m\_lock = a\_lock;

m\_startday = a\_start;

m\_first\_year = a\_first\_year;

m\_next\_tov=a\_nextcrop;

}

---

## Member Data Documentation

TTypesOfVegetation FarmEvent::m\_event

Referenced by FarmEvent(), and Farm::LeSwitch().

LE\* FarmEvent::m\_field

Referenced by Farm::CheckRotationManagementLoop(), FarmEvent(), Farm::HandleEvents(), and Farm::LeSwitch().

bool FarmEvent::m\_first\_year

Referenced by FarmEvent().

bool FarmEvent::m\_lock

Referenced by FarmEvent().

TTypesOfVegetation FarmEvent::m\_next\_tov

Referenced by FarmEvent().

long FarmEvent::m\_run

Referenced by FarmEvent(), and Farm::LeSwitch().

int FarmEvent::m\_startday

Referenced by FarmEvent().

int FarmEvent::m\_todo

Referenced by FarmEvent(), and Farm::LeSwitch().

---

The documentation for this struct was generated from the following file:

- farm.h


- FarmEvent
- Generated on Thu Jan 10 2013 13:15:36 for ALMaSS Skylark ODdox by
   1.8.1.1
